# Effects of alcohol intoxication on driving performance, confidence in driving ability, and psychomotor function: a randomized, double-blind, placebo-controlled study

**DOI:** 10.1007/s00213-022-06260-z

**Published:** 2022-11-02

**Authors:** Harriet Garrisson, Andrew Scholey, Joris C. Verster, Brook Shiferaw, Sarah Benson

**Affiliations:** 1 Centre for Human Psychopharmacology, Faculty of Health, Arts and Design, Swinburne University of Technology, Hawthorn, VIC 3122 Australia; 2 Nutrition Dietetics and Food, School of Clinical Sciences, Monash University, Melbourne, VIC Australia; 3 Division of Pharmacology, Utrecht Institute for Pharmaceutical Sciences, Utrecht University, Utrecht, Netherlands; 4 Seeing Machines Ltd, ACT, Canberra, Australia

**Keywords:** Alcohol, Driving performance, Simulator, Psychomotor function, Road safety, Subjective awareness

## Abstract

**Rationale:**

Alcohol-induced driving impairment can occur with any departure from a zero-blood alcohol concentration (BAC). Because intoxication is characterised by impaired judgement, drivers under the influence of alcohol may overestimate their capacity to safely operate a vehicle.

**Objectives:**

This study examined the effects of alcohol on driving performance, four-choice reaction time (FCRT), and self-rated confidence in driving ability. It specifically focused on alcohol doses equal to commonly enforced legal BAC limits (i.e. 0.05% and 0.08%).

**Methods:**

A randomized, double-blind, placebo-controlled design was utilised. Seventeen participants were tested in three conditions: placebo and two alcohol conditions aiming for BACs of 0.05% and 0.08%. Participants underwent a baseline FCRT task and a 1-h simulated highway driving task before completing another FCRT task and rated their confidence in their driving ability.

**Results:**

The high and low alcohol dose conditions resulted in a mean BAC of 0.07%, and 0.04%, respectively (*n* = 17). The high BAC treatment significantly increased standard deviation of lateral position (SDLP) by 4.06 ± 5.21 cm and standard deviation of speed (SDS) by 0.69 ± 0.17 km/h relative to placebo, while confidence in driving ability remained unchanged across treatments. FCRT performance was impaired by the high BAC treatment (all < 0.01), but there we no significant differences between placebo and low BAC conditions.

**Conclusions:**

The findings of this study show that driving performance and associated psychomotor functioning become significantly impaired below legally permissible driving limits in some jurisdictions. We identified a dissociation between driving performance and subjective awareness of impairment. Despite a significantly diminished driving ability at 0.07% BAC, drivers were unaware of their impairment.

## Introduction

Road traffic collisions (RTCs) cause more than 3700 fatalities globally per day, with up to 35% being alcohol-related (World Health Organization et al. 2018). Alcohol intoxication manifests in brain areas responsible for higher level functioning (Ogden and Moskowitz 2004) and consequently impairs several aspects of cognition required for safe driving (Breitmeier et al. 2007; Jongen et al. 2014). Past research has repeatedly demonstrated that a driver’s risk of collision increases exponentially with a rising blood alcohol concentration (BAC), and the odds of that incident resulting in serious injury or death is approximately 3.5 times higher for drink-drivers than sober drivers (Ogden and Moskowitz 2004; Traynor 2005). Despite this, many jurisdictions permit fully licensed drivers to operate a vehicle while under the influence of alcohol, with most enforcing legal BAC limits of 0.05% (e.g. Australia and New Zealand) or 0.08% (e.g. England and most states within the USA). While significant progress has been made towards strengthening existing legislation that penalizes driving under the influence of alcohol, drink-drivers remain over-represented in road trauma cases (World Health Organization 2018).

Modern-day driving simulators provide an opportunity to investigate alcohol-related deficits in driving performance within a controlled and relatively realistic environment. Measures of driving performance often include parameters such as speed (variability), reaction time, steering behaviour, excursions out of lane, number of collisions, and in particular the standard deviation of lateral position (SDLP), or “weaving” of the car (Irwin et al. 2017; Verster and Roth 2014, 2011). A reduction in these abilities may ultimately result in an increased likelihood of collision. For instance, an increase in SDLP may result in lane crossing into an adjacent oncoming traffic lane (Owens and Ramaekers 2009; Verster and Roth 2014). Studies indicate that a driver’s SDLP may become compromised at a BAC as low as 0.021%, increasing in a dose-dependent manner by 0.7 cm for every 0.01% BAC increase thereafter, with significant increases in SDLP occurring at BAC levels above 0.05% (Irwin et al. 2017; Mets et al. 2011). A commonly used driving simulator assessment is the 100-km highway drive, adopted from on-the-road studies (Verster and Roth 2011). Sufficient time on task is essential to uncover any potential adverse effects of alcohol on driving ability. Vigilance decrement is an important feature of driving assessment tasks, as drivers may put forth extra effort at the start of a task, thereby reducing the impairing effects of alcohol. As the task continues, it becomes increasingly difficult to counteract such impairing effects (Verster and Roth 2013). Although SDLP is sensitive to the effects of alcohol, lane-keeping performance does not encompass other higher-order demands in driving, such as route-finding and interactions with other traffic.

Driving is a complex behaviour requiring a combination of psychomotor and perceptual skills, including the ability to detect, analyse and respond to incoming information, maintain attention, and coordinate physiological movement (Anstey et al. 2005; Chamberlain and Solomon 2002). As such, lab-based cognitive assessments are often administered to assess the effects of alcohol on specific aspects of driving performance (Garrisson et al. 2021). One such test that has shown robust sensitivity to the impairing effects of alcohol at moderate to high BAC levels and is a purported alcohol-sensitive, driving-relevant psychomotor task is the four-choice reaction time (FCRT) task. The FCRT task is a complex reaction time (CRT) task that requires participants to press appropriate response buttons corresponding to one of four highlighted stimuli. Research has demonstrated that during CRT tasks, participants under the influence of alcohol tend to make more errors, while response speed remains unchanged relative to placebo (Mackay et al. 2002; Tiplady et al. 2001, 2004). Consistent with the alcohol myopia theory proposed by Josephs and Steele (1990), alcohol reduces attentional capacity, with diminished resources causing an individual to prioritize only the most salient aspects of a situation while neglecting less important aspects. While driving, an individual is required to continuously process and react to novel situations (Jongen et al. 2016). An intoxicated driver, however, may subconsciously prioritise certain aspects of driving over others, leading to more errors and unsafe driving that increases the likelihood of accidents (Tiplady et al. 2001).

Beyond psychomotor disturbances, alcohol is commonly associated with impaired judgement and greater risk-taking behaviour (Burian et al. 2002; Tiplady et al. 2004). In the context of driving, having an accurate perception of one’s own ability, particularly following alcohol consumption, is essential to take appropriate countermeasures to mitigate risk, for example, choosing not to drive when impaired (Verster and Roth 2012). Even when sober, drivers tend to demonstrate poor judgement of their own driving performance and evaluate their own driving as better and less risky than the average driver (Jones et al. 2006; McCormick et al. 1986; Svenson 1981). Because drinkers tend to underestimate their BAC (Fillmore and Blackburn 2002; Köchling et al. 2021), they are likely to make erroneous judgements regarding their ability to drive safely and mistakenly engage in risky or dangerous behaviour (Marczinski and Fillmore 2009; Köchling et al. 2021). Previous research has yielded mixed results relating to self-evaluated driving performance after alcohol consumption. On the one hand, individuals are unable to accurately judge changing in their own driving performance at a BAC of 0.05% (Verster and Roth 2011, 2012), while at higher BACs (i.e. 0.081% to 0.09%), individuals accurately appraised their driving as impaired (Harrison and Fillmore 2011). When an additional divided attention task was added, however, participants were no longer able to recognise performance deficits, as the distraction exacerbated impairment (Harrison and Fillmore 2011). The idea that alcohol may produce objective impairment that does not match subjective impairment holds significant implications in traffic safety, education programmes, and in particular warnings about drink-driving, as the onus is on the driver to decide if they are competent to drive. Given that the links between subjective intoxication and alcohol induced performance deficits have received only limited investigation, it is important to investigate whether subjective awareness parallels objective indices of cognitive and behavioural impairment as it relates to driving.

The current study was conducted to expand on existing evidence by investigating the effects of alcohol on sustained simulated driving and psychomotor performance. The primary objective of this study was to examine the effects of alcohol at BAC levels of 0.05% and 0.08% on a 1-h simulated highway drive and FCRT. These BAC levels were selected based on commonly enforced on-road drink-driving limits in most countries. A secondary objective was to examine the association between objective driving ability and subjective awareness of driving impairment to determine whether intoxicated drivers are able to accurately assess their driving ability.

## Method

### Design

This study utilized a randomized, double-blind, placebo-controlled, crossover design whereby participants underwent three conditions (placebo; BAC 0.05%; BAC 0.08%) in counterbalanced order on separate visits before completing objective and subjective measures of driving performance and psychomotor function. The current study was run between 2014 and 2019 and was approved by the Swinburne University Human Research Ethics Committee (SUHREC, approval number 2014/065) and was conducted in accordance with the Declaration of Helsinki.

### Participants

Seventeen participants, including 8 males and 9 females, with a mean age of 24.94 ± 3.09 years (range = 20 to 30) completed the study. Participants were eligible for the study if they were 18 to 40 years old, had normal or corrected to normal vision, held a valid driver’s license, regularly consumed alcohol (at least once monthly), and weighed under 100 kg. Participants with a known history of psychiatric, neurological, or medical conditions were excluded, as were those susceptible to simulator sickness (determined using the Simulator Sickness Questionnaire (SSQ; Kennedy et al. 1993)). Additional exclusion criteria included current or past alcohol abuse (as determined by the Alcohol Use Disorders Identification Test (AUDIT; Babor et al. 2001)), smokers, current use of psychoactive medication expected to interfere with performance on any of the measures and females who were pregnant, and potentially pregnant or lactating.

### Procedure

Participants were required to attend a screening visit and three testing visits at Swinburne University of Technology Centre for Human Psychopharmacology in Melbourne, Australia. During the screening visit, participants provided written informed consent before being assessed for eligibility and familiarizing themselves with the procedure. The randomization of the order of treatments for each participant was determined using computer generated and blocked randomization by a disinterested third party and kept on a password-protected spreadsheet that was not accessed by the investigator during data collection. All treatments were prepared away from the investigator and participant by the research nurse. On study days, participants were asked to refrain from food for 2 h prior to testing, products containing caffeine within 12 h of testing, and alcohol within 24 h of testing and were asked to consume the same breakfast on each testing day.

**Fig. 1:**
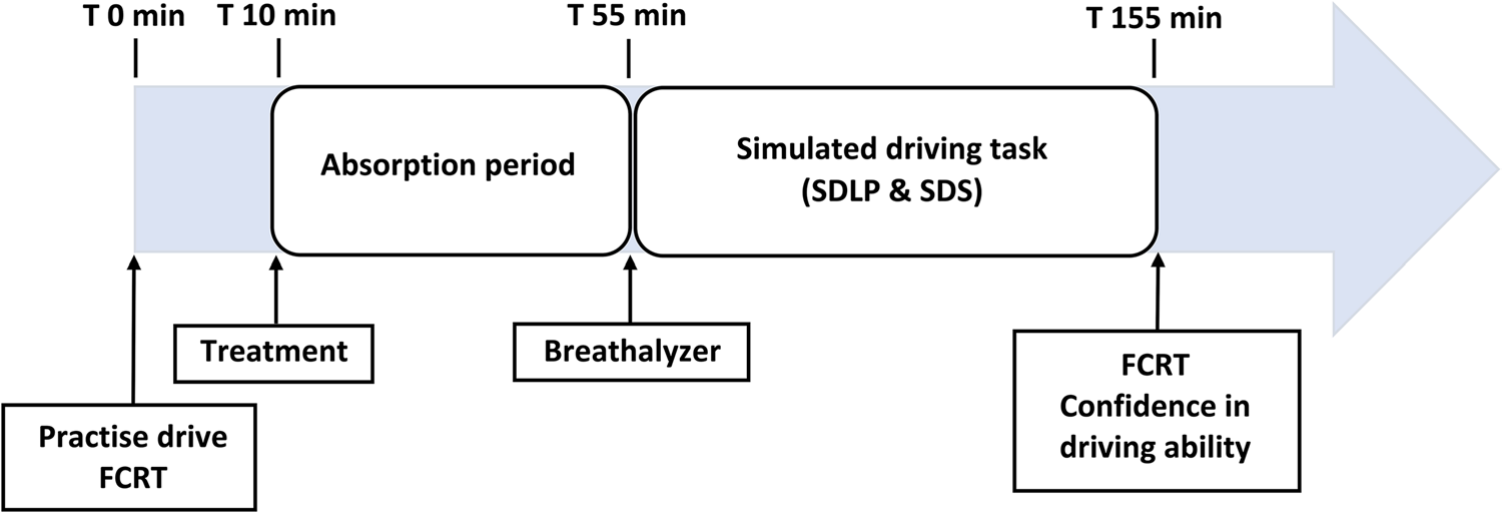
Experimental procedure

Participants arrived at the testing site between 9:00 AM and 11:00 AM. All participants were breathalysed at the beginning of their testing sessions to confirm a zero BAC. Prior to treatment, participants completed a 10-min practice drive on the simulator and a baseline measure of the FCRT task. Participants were administered that day’s drink and asked to consume their beverage at a steady pace over the first 10 min of the 45-min absorption period. This timing was based on the average *t*_max_ (the time for the drug to reach maximum concentration in the blood) of alcohol, which is generally 30–45 min (Saunders and Rey 2011). A second BAC reading was taken at 45 min post treatment, followed by a 105-km simulated highway drive, lasting approximately 1 h. Following the driving test, participants completed the FCRT task again and were asked to rate their confidence in their driving ability. Participants were administered a final breathalyser to confirm a < 0.05% BAC before leaving the testing site. The experimental procedure is visualised in Fig. 1.

### Treatment

To meet the target BACs of 0.05% or 0.08% at 45 min post-consumption, participants were administered either 0.6 g/kg or 0.85 g/kg of Absolut vodka (40% alcohol by volume) mixed with 250 ml of orange juice, respectively. Participants were asked to consume their beverage at a steady pace over a 10-min period with a 35-min absorption period following. Participants assigned to the placebo condition were administered a glass of orange juice with vodka rubbed around the rim for the purpose of blinding. All treatments were prepared by the nurse away from the participant and investigator.

### Blood alcohol concentration (BAC)

Breathalyser readings were taken with a frequently calibrated Victorian Police Lion Alcolmeter SD-400PA breathalyser, which measured Breath Alcohol Concentration (BrAC) as grams of alcohol per 210 L of breath.

### Performance measures

#### Driving performance

**Fig. 2:**
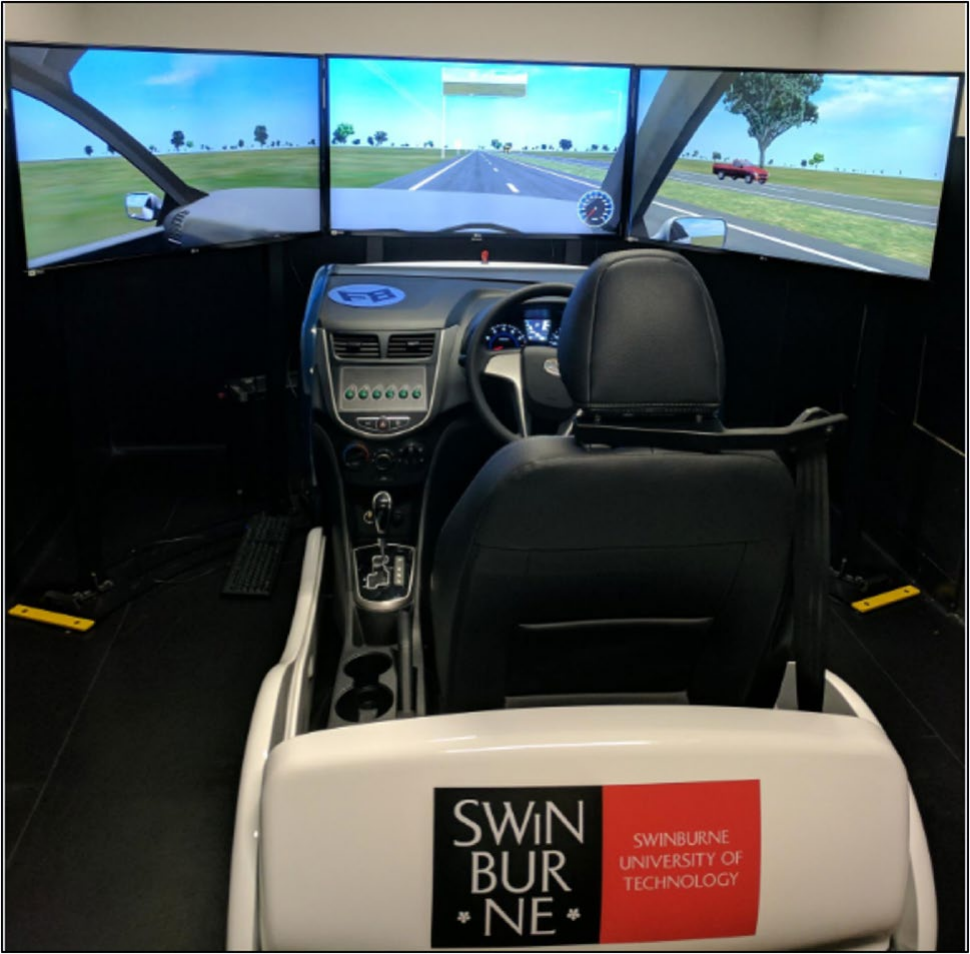
The Forum8 driving simulator and displays

Driving performance was assessed using a Forum8 medium-fidelity driving simulator (Fig. 2) which incorporated the simulation software UC-Win/ROAD version 11 (Forum8 Co., Ltd.).

The mock-up car unit consisted of an adjustable car seat, a seat belt, dashboard, steering wheel, turn sign indicators, a gear lever, and brake and accelerator pedals for vehicle control. The simulation was presented on a wide view display made up of three 42″ LCD flat panel monitors positioned side by side, each with a resolution of 1920 × 1080. Auditory feedback included the sound of the engine, braking, accelerating, and driving off-road. The 105-km highway driving scenario developed by Forum8 AU Pty Ltd resembled the Princes Highway, Victoria, and was tailored to Australian traffic situations, including common traffic signs, vehicles, and scenery.

Participants were instructed to drive within the left lane maintaining steady lateral position and a constant speed of 100 km/h. Participants were required to occasionally overtake slower-driving cars. All data collected prior to the car reaching a speed of 60 km/h, in addition to extreme values for lane curvature, were removed prior to the analysis. Overtaking actions were marked by the participant’s use of the indicator and removed before data analysis.

Driving performance was operationalised as SDLP and SDS. SDLP represents the deviation (standard deviation) from the mean lateral position of the car within the left traffic lane (Verster and Roth 2011). SDS represents the standard deviation of speed. SDLP is the most reported measure of driving performance in the literature and has been shown to correlate with BAC level (Irwin et al. 2017). Although one outcome measure (overall mean) is often presented for the entire 100-km drive, a typical characteristic of the 100-km driving test is a vigilance decrement. Specifically, SDLP values increase over the duration of the driving task (Verster and Roth 2013, 2011). As such, SDLP calculated for shorter successive segments can illustrate this performance decrement, in addition to overall SDLP.

#### Four-choice reaction time

**Fig. 3:**
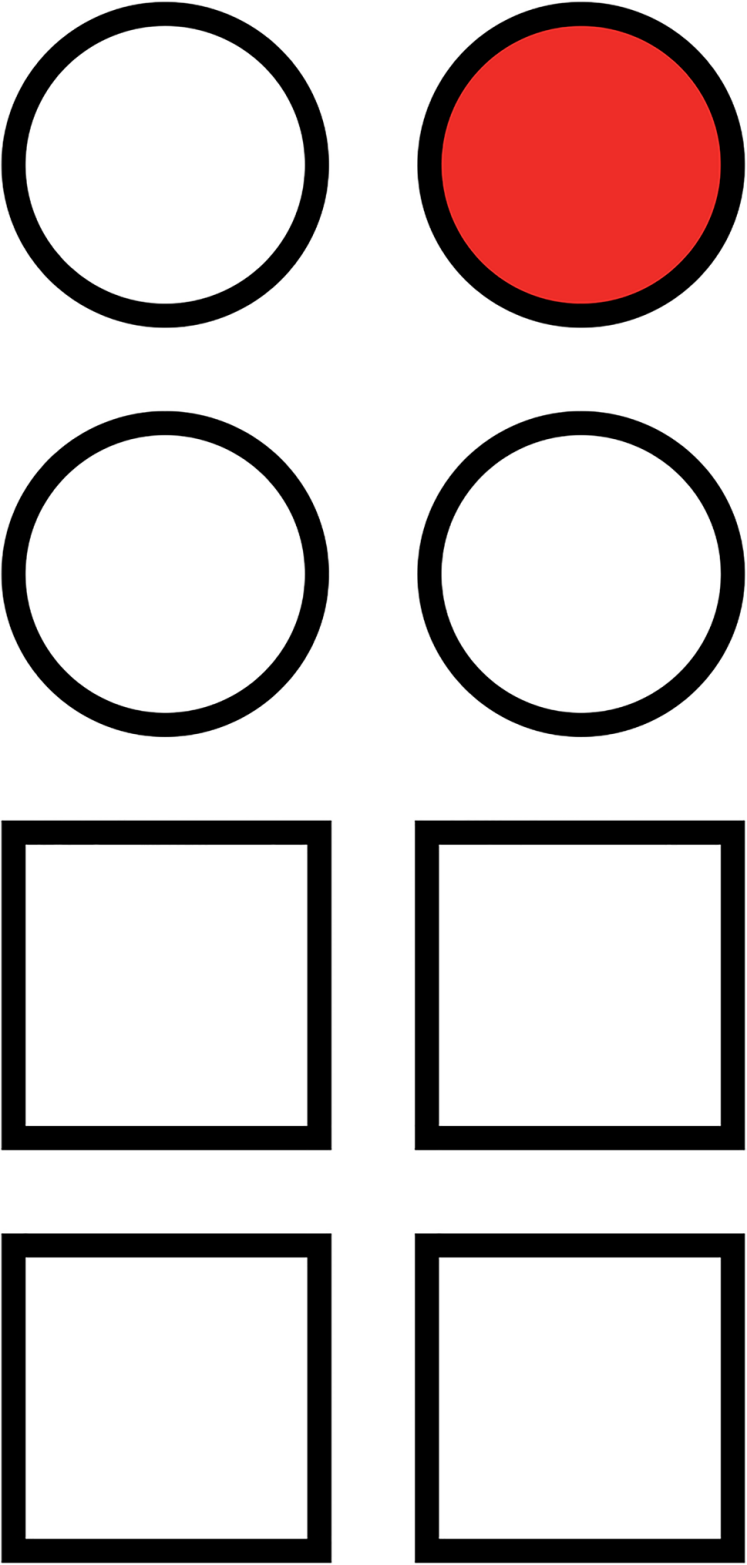
FCRT task showing the relative position of stimuli (circles) and response (squares). In the example, the correct response is the top right square

Complex RT and psychomotor performance were evaluated as purported alcohol-sensitive, driving-relevant cognitive skills. The FCRT task (Tiplady et al. 2001) consisted of 80 trials and was completed on a touchpad device displaying a two-by-two stimulus array of four circles corresponding to a two-by-two response array of four squares. The circles were coloured red one at a time, and participants were required to tap the corresponding square as quickly and accurately as possible (see Fig. 3). The stimuli were presented in a random sequence, whereby both RT and errors were recorded.

#### Confidence in driving performance

Immediately following each driving test, subjects indicated their perceived quality of driving on a 100 mm VAS ranging from 0 (‘I drove exceptionally poor’) to 20 (‘I drove exceptionally well’), with the midpoint indicating normal performance (‘I drove normally’) (Verster and Roth 2011).

### Statistical analysis

Statistical analyses were performed using IBM SPSS Statistical Software, Version 25.0 (SPSS Inc. Chicago, IL). There was no pre-defined statistical analysis plan. Prior to the main analysis, distribution of the data was assessed using the Shapiro–Wilk test. As FCRT data was non-normally distributed (*p* values < 0.05), data analyses comparing treatment data to corresponding baseline data were performed using a Wilcoxon signed-rank test. Repeated-measures analyses of variance (ANOVA) tests were used to examine the effect of treatment (placebo, low BAC and high BAC) on driving outcomes (SDLP and SDS) and confidence in driving performance. An additional repeated-mores ANOVA was conducted to examine changes in SDLP over time (grouped in 20-min increments) following placebo, low BAC, and high BAC treatments. Follow-up Bonferroni-corrected post hoc pairwise comparisons were conducted to assess specific differences between conditions (revised α = 0.05/3 = 0.017, two-sided). All other analyses were conducted as two-sided and *p* values < 0.05 were considered significant unless stated otherwise.

## Results

### BAC level

**Fig. 4:**
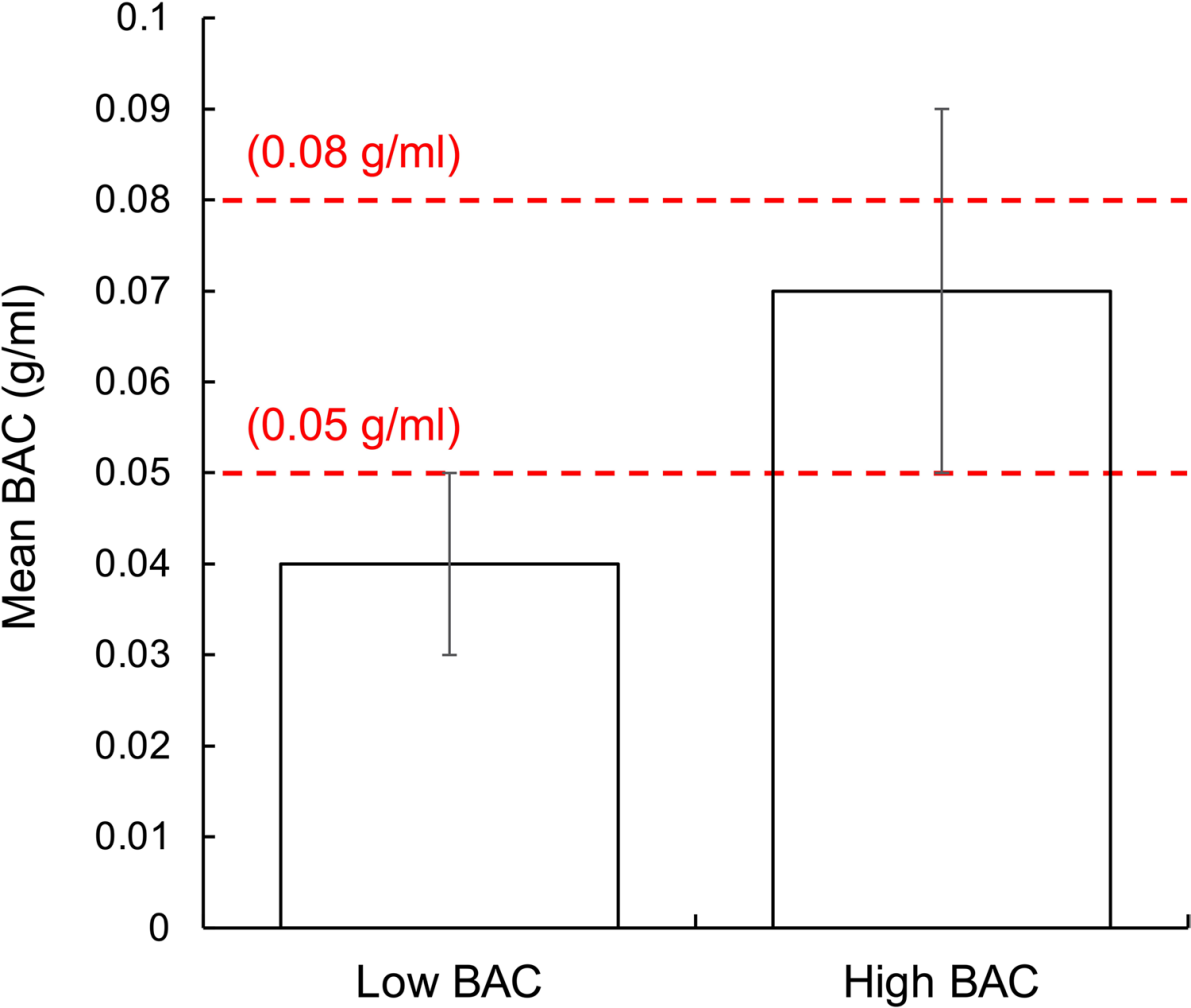
Mean BACs and standard deviations for low and high BAC groups. The red lines indicate legal driving limits in several jurisdictions (World Health Organization 2018)

The average BAC profiles for both alcohol treatments are shown in Fig. 4.

As shown in Fig. 4, participants reached a mean BAC level of 0.04 ± 0.01 g/ml (range: 0.03 to 0.05) and 0.07 ± 0.02 g/ml (range: 0.05 to 0.09) for the low and high BAC treatments, respectively.

### Driving performance

The results of the repeated-measure ANOVAs for both driving outcomes and subjective ratings of confidence in driving performance across treatments are shown in Table 1.

**Table 1:** Mean SDLP, SDS and confidence in driving performance scores and standard deviations across treatments

Outcome	Placebo	Low BAC	High BAC
SDLP (cm)	21.09 ± 7.06^*^	22.03 ± 6.37	25.15 ± 5.80^*^
SDS (km/h)	2.39 ± 1.06	2.68 ± 1.21	2.76 ± 1.18
Confidence	57.91 ± 24.16	54.94 ± 19.36	54.56 ± 16.59

*N* = 17; all values expressed as mean ± standard deviation; SDLP = standard deviation of lateral position; SDS = standard deviation of speed; confidence = confidence in driving performance (expressed as a score ranging 0 to 100 with higher scores reflecting greater confidence in driving performance); ^*^significant difference between conditions at *p* < 0.017 (Bonferroni correction)

**Fig. 5:**
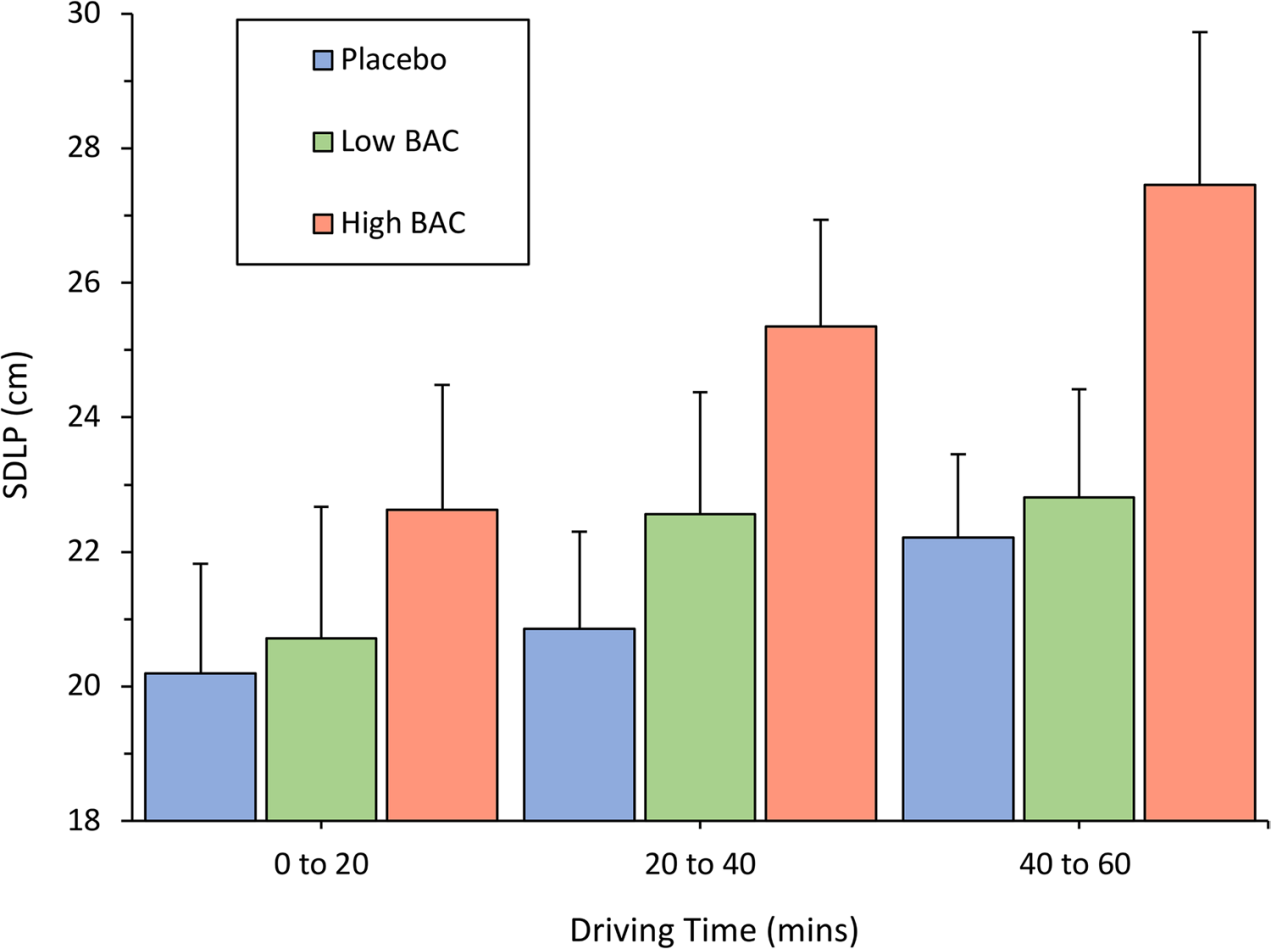
Mean SDLP and standard error of the mean in 20-min increments across treatments

As shown in Table 1, there were significant main effects of treatment for both SDLP [F(2,32) = 7.66, *p* = 0.002, η_p_^2^ = 0.324] and [SDS: F(2,32) = 3.80, *p* = 0.033, η_p_^2^ = 0.192]. Post hoc analysis with a Bonferroni correction revealed a significant increase in SDLP from placebo to the high BAC condition (*p* = 0.016) and from the low to high BAC conditions (*p* = 0.004). There were no significant differences in SDLP between the placebo and low BAC conditions, or in SDS across conditions (all *p* > 0.05). Driving performance was further investigated by examining SDLP in 20-min increments (Fig. 5).

As shown in Fig. 5, SDLP did not significantly increase over time following the placebo treatment (*p* = 0.168). Following both alcohol treatments, however, there was a main effect of time for SDLP [low BAC: F(2,32) = 3.70, *p* = 0.036, η_p_^2^ = 0.638; high BAC: F(2,32) = 3.50, *p* = 0.042, η_p_^2^ = 0.180]. A series of post hoc analyses with Bonferroni correction revealed that SDLP was greater at the second time point relative to the first time point in the high BAC condition (*p* = 0.002). There were no differences between the time points in the low BAC condition, or between the first and third time point, and second and third time points in the high BAC condition (all *p* > 0.017).

### Confidence in driving performance

There were no significant differences in confidence in driving performance across conditions (*p* = 0.819) (Table 1).

### Four-choice reaction time

The results of the CRT task are shown in Table 2.

**Table 2:** Median FCRT task scores and quartile ranges at baseline and post treatment across conditions

Outcome	Placebo			Low BAC			High BAC		
	Baseline	Post	*p*	Baseline	Post	*p*	Baseline	Post	*p*
RT	391.50 (371.50–414.75)	390.50 (373.00–413.25)	0.720	394.00 (377.25–419.00)	401.50 (378.00–431.75)	0.387	396.50 (375.50–429.25)	423.00 (409.25–443.75)	< 0.001^*^
Errors	1.00 (0–1.75)	1.19 ± 1.38	0.464	0.5 (0–2.00)	1.00 (0–2.00)	0.728	0 (0–1.00)	1.00 (1.00–3.00)	0.001*

*N* = 16; all values expressed as *median (interquartile range)*; RT = reaction time (ms); post = post treatment; *p < 0.05

Because of missing FCRT data for one participant in the high BAC condition, median FCRT scores were calculated based on *n* = 16. There were no significant differences in RT or errors in the placebo and low BAC conditions (all *p* > 0.05). Following the high BAC treatment, however, both RT [Z = -3.03, *p* = 0.002] and errors [Z = -2.97, *p* = 0.003] increased relative to baseline.

## Discussion

The current study investigated the effects of alcohol on driving performance, psychomotor functioning, and confidence in driving ability. Breath analysis prior to the simulated driving task revealed average BACs of 0.04% and 0.07% for the low and high conditions, respectively. As such, the low and high BAC conditions were just below two common legal thresholds for driving. The results confirmed that alcohol at a BAC of 0.07% significantly impaired driving performance, represented by a 4.06 cm increase in SDLP. These findings are in-line with those of a previous systematic review demonstrating that acute alcohol consumption resulted in an increased SDLP at BAC levels above 0.05% (Irwin et al. 2017). A reduction in the capacity to control speed and deviation of a vehicle may ultimately result in an increased likelihood of collision, for example, an increase in SDLP may result in lane crossing into an adjacent traffic lane*.* Other research linking BAC > 0.05% to increased crash risk lends support to a legally enforceable drink-driving limit of a 0.05% BAC (Borkenstein et al. 1964). As we did not specifically measure collision risk, real-world translation of these findings is limited.

SDLP did not significantly increase over time following the placebo treatment; however, following both alcohol treatments (low and high), SDLP increased significantly from the first 20 min onwards as time and distance driven increased, indicating that alcohol significantly compounded the deficits of driver performance. As no change in SDLP over time occurred in the placebo condition, this indicates that the addition of alcohol was the primary factor contributing to performance decrements, above any decrements resulting from time on task. Although BAC was not recorded throughout the drive, given it was approximately 1 h in duration, it is likely that performance declined over time as participants moved into the descending limb of intoxication and experienced vigilance decrement. Previous research examining driving-related behaviour has predominately focused on alcohols effects on the ascending phase of alcohol intoxication; however, the descending phase may be of particular importance as driving ability has been found to deteriorate substantially on the descending limb of intoxication (Fillmore and Blackburn 2002; Weafer and Fillmore 2012). In fact, a review of 26 articles found that participants made twice as many errors during simulated driving on the descending limb, compared with the ascending limb (Holland and Ferner 2017). Furthermore, it has been posited that intoxicated drivers often make an initial effort to compensate for some of the impairing effects of alcohol (Fillmore and Blackburn 2002); however, as the driving task required sustained vigilance, it is likely that participants began to experience fatigue, resulting in a reduced capacity to maintain alertness and focused attention on the driving task (Verster and Roth 2013).

With respect to the effect of alcohol on FCRT performance, both RT and errors increased in the high BAC condition relative to placebo. Contrary to expectations, there was no evidence of an alcohol-related speed-accuracy trade-off during the FCRT task. This is inconsistent with previous research that has indicated at BACs ranging from 0.066% to 0.08%, a speed-accuracy trade-off occurred whereby participants made less accurate responses but showed no impairment in reaction speed (Mackay et al. 2002; Tiplady et al. 2001, 2004). This may be in part because in the FCRT task used in the aforementioned studies, some of the time the sequence of stimuli was random, while other times the same sequence was repeated. Despite the absence of a speed-accuracy trade-off, the finding that CRT performance is impaired at a BAC of 0.07% is consistent with the previous research that also found performance deficits at this level.

Although there was evidence of impairment to driving and cognitive performance following the high BAC treatment relative to placebo, participant’ confidence in their driving ability remained unchanged. These findings demonstrate a dissociation between actual performance and subjective ratings thereof. A possible rationalisation that may contribute to drinkers’ over-confidence is the development of acute tolerance. When acute tolerance occurs, the effects of alcohol feel greater immediately following alcohol consumption and subside over time, even if BACs are comparable. This often results in a diminished intensity of subjective impairment during the descending limb, compared to the ascending limb (Marczinski and Fillmore 2009). As confidence measures were obtained after the completion of the driving task, sometime after alcohol administration, it is likely that participants were in the descending limb of intoxication, hence experiencing acute tolerance to the effects of the ingested alcohol and appraising their performance as unaffected. The capacity of an individual to recognise subsequent impairments in driving abilities following alcohol consumption hold significant implications in traffic safety, as it is generally the driver’s responsibility to decide if they are competent to drive. These findings demonstrate that a driver’s perception of the situation may not reflect objective reality. Even if people feel confident in their driving performance while intoxicated, their driving performance may be considerably deteriorated and unsafe.

While this study holds valuable implications, it is not without limitations. The first limitation of the current study was the relatively small sample size. It is therefore difficult to draw definitive conclusions from the data, and in instances where no effect or limited differences were observed, the issue of power and possibility of type II errors should be considered. Additionally, we reported a large treatment effect (η_p_^2^ = 0.192) for SDLP and a large effect of time on SDLP during the high BAC condition (η_p_^2^ = 0.180). Due to the small sample size, however, the reliability of any observed effect sizes is questionable and should be interpreted with caution. Another limitation of the current study is the delay between BAC measurement and self-assessment, as this is enough time for BAC levels to increase or decrease from the initial level. It may be useful for future researchers to ask participants to predict their driving performance prior to driving test to further understand how alcohol impacts on their perception of their driving skills. Moreover, simulator-based driving tests are not fully representative of the conditions or risks present in real-world driving. It is likely that drivers would behave differently when presented with actual traffic given the increased consequences following inaccurate decisions. Additionally, the absence of actual consequences of crashes such as injury or death which are real in on-road driving may have had an impact on subjective measures of confidence in driving ability. As such, these findings should be interpreted with caution, and on-road validation in actual traffic is required to confirm the reliability of the results. Lastly, it is important to acknowledge that a potential RTC could result from as little as one bad deviation from the road. As such, future research may benefit from analysing lane crossing and/or extreme SDLP values using the survival analysis technique to provide additional insight into collision risk among intoxicated drivers.

In conclusion, our study confirmed previous findings that clinically significant impairment in driving performance and psychomotor functioning was evident at a BAC of 0.07%. This BAC is above the legal limit for driving of 0.05%, enforced in most countries around the world (World Health Organization et al. 2018), although in some countries, such as England and parts of the USA, a legal limit of 0.08% is enforced. Of importance, our results showed that the objective deficits in driving performance occurred in the absence of a concomitant reduction in self-reported ability. Indeed, participants in the current study showed a poor ability to judge their own driving performance, rating it as being relatively consistent across the treatment conditions. This observation is particularly important as the vast majority of countries around the world enforce BAC 0.05% as legal limit for driving. It is important for future research to continue to establish how specific driving-related cognitive skills are impacted by alcohol and investigate possible discrepancies between objective and subjective measures of impairment, specifically at legally permissible BAC limits.
